# Data visualisation in scoping reviews and evidence maps on health topics: a cross-sectional analysis

**DOI:** 10.1186/s13643-023-02309-y

**Published:** 2023-08-17

**Authors:** Emily South, Mark Rodgers

**Affiliations:** Centre for Reviews and Dissemination, University of York, York, YO10 5DD UK

**Keywords:** Scoping review, Evidence map, Data visualisation

## Abstract

**Background:**

Scoping reviews and evidence maps are forms of evidence synthesis that aim to map the available literature on a topic and are well-suited to visual presentation of results. A range of data visualisation methods and interactive data visualisation tools exist that may make scoping reviews more useful to knowledge users. The aim of this study was to explore the use of data visualisation in a sample of recent scoping reviews and evidence maps on health topics, with a particular focus on interactive data visualisation.

**Methods:**

Ovid MEDLINE ALL was searched for recent scoping reviews and evidence maps (June 2020-May 2021), and a sample of 300 papers that met basic selection criteria was taken. Data were extracted on the aim of each review and the use of data visualisation, including types of data visualisation used, variables presented and the use of interactivity. Descriptive data analysis was undertaken of the 238 reviews that aimed to map evidence.

**Results:**

Of the 238 scoping reviews or evidence maps in our analysis, around one-third (37.8%) included some form of data visualisation. Thirty-five different types of data visualisation were used across this sample, although most data visualisations identified were simple bar charts (standard, stacked or multi-set), pie charts or cross-tabulations (60.8%). Most data visualisations presented a single variable (64.4%) or two variables (26.1%). Almost a third of the reviews that used data visualisation did not use any colour (28.9%). Only two reviews presented interactive data visualisation, and few reported the software used to create visualisations.

**Conclusions:**

Data visualisation is currently underused by scoping review authors. In particular, there is potential for much greater use of more innovative forms of data visualisation and interactive data visualisation. Where more innovative data visualisation is used, scoping reviews have made use of a wide range of different methods. Increased use of these more engaging visualisations may make scoping reviews more useful for a range of stakeholders.

**Supplementary Information:**

The online version contains supplementary material available at 10.1186/s13643-023-02309-y.

## Background

Scoping reviews are “a type of evidence synthesis that aims to systematically identify and map the breadth of evidence available on a particular topic, field, concept, or issue” ([1], p. 950). While they include some of the same steps as a systematic review, such as systematic searches and the use of predetermined eligibility criteria, scoping reviews often address broader research questions and do not typically involve the quality appraisal of studies or synthesis of data [2]. Reasons for conducting a scoping review include the following: to map types of evidence available, to explore research design and conduct, to clarify concepts or definitions and to map characteristics or factors related to a concept [3]. Scoping reviews can also be undertaken to inform a future systematic review (e.g. to assure authors there will be adequate studies) or to identify knowledge gaps [3]. Other evidence synthesis approaches with similar aims have been described as evidence maps, mapping reviews or systematic maps [4]. While this terminology is used inconsistently, evidence maps can be used to identify evidence gaps and present them in a user-friendly (and often visual) way [5].

Scoping reviews are often targeted to an audience of healthcare professionals or policy-makers [6], suggesting that it is important to present results in a user-friendly and informative way. Until recently, there was little guidance on how to present the findings of scoping reviews. In recent literature, there has been some discussion of the importance of clearly presenting data for the intended audience of a scoping review, with creative and innovative use of visual methods if appropriate [7–9]. Lockwood et al. suggest that innovative visual presentation should be considered over dense sections of text or long tables in many cases [8]. Khalil et al. suggest that inspiration could be drawn from the field of data visualisation [7]. JBI guidance on scoping reviews recommends that reviewers carefully consider the best format for presenting data at the protocol development stage and provides a number of examples of possible methods [10].

Interactive resources are another option for presentation in scoping reviews [9]. Researchers without the relevant programming skills can now use several online platforms (such as Tableau [11] and Flourish [12]) to create interactive data visualisations. The benefits of using interactive visualisation in research include the ability to easily present more than two variables [13] and increased engagement of users [14]. Unlike static graphs, interactive visualisations can allow users to view hierarchical data at different levels, exploring both the “big picture” and looking in more detail ([15], p. 291). Interactive visualizations are often targeted at practitioners and decision-makers [13], and there is some evidence from qualitative research that they are valued by policy-makers [16–18].

Given their focus on mapping evidence, we believe that scoping reviews are particularly well-suited to visually presenting data and the use of interactive data visualisation tools. However, it is unknown how many recent scoping reviews visually map data or which types of data visualisation are used. The aim of this study was to explore the use of data visualisation methods in a large sample of recent scoping reviews and evidence maps on health topics. In particular, we were interested in the extent to which these forms of synthesis use any form of interactive data visualisation.

## Methods

This study was a cross-sectional analysis of studies labelled as scoping reviews or evidence maps (or synonyms of these terms) in the title or abstract.

### Search

The search strategy was developed with help from an information specialist. Ovid MEDLINE® ALL was searched in June 2021 for studies added to the database in the previous 12 months. The search was limited to English language studies only.

The search strategy was as follows:

Ovid MEDLINE(R) ALL

1. (scoping review or evidence map or systematic map or mapping review or scoping study or scoping project or scoping exercise or literature mapping or evidence mapping or systematic mapping or literature scoping or evidence gap map).ab,ti.
2. limit 1 to english language
3. (202006* or 202007* or 202008* or 202009* or 202010* or 202011* or 202012* or 202101* or 202102* or 202103* or 202104* or 202105*).dt.
4. 2 and 3

The search returned 3686 records. Records were de-duplicated in EndNote 20 software, leaving 3627 unique records.

### Sampling

A sample of these reviews was taken by screening the search results against basic selection criteria (Table 1). These criteria were piloted and refined after discussion between the two researchers. A single researcher (E.S.) screened the records in EPPI-Reviewer Web software using the machine-learning priority screening function. Where a second opinion was needed, decisions were checked by a second researcher (M.R.).

**Table 1:** Selection criteria

Include	Exclude
Described with one of these terms in title or abstract: scoping review, evidence map, systematic map, mapping review, scoping study, scoping project, scoping exercise, literature mapping, evidence mapping, systematic mapping, literature scoping, evidence gap map	Full systematic review and/or meta-analysis
Meets basic criteria for a review (systematic search and inclusion criteria)	Protocol for a review
Review of empirical evidence	Review of guidance, legislation, tools, etc
Related to human health (including wider determinants)	Conference abstract
	Not in English
	Cannot access full text through institutional access

Our initial plan for sampling, informed by pilot searching, was to screen and data extract records in batches of 50 included reviews at a time. We planned to stop screening when a batch of 50 reviews had been extracted that included no new types of data visualisation or after screening time had reached 2 days. However, once data extraction was underway, we found the sample to be richer in terms of data visualisation than anticipated. After the inclusion of 300 reviews, we took the decision to end screening in order to ensure the study was manageable.

### Data extraction

A data extraction form was developed in EPPI-Reviewer Web, piloted on 50 reviews and refined. Data were extracted by one researcher (E. S. or M. R.), with a second researcher (M. R. or E. S.) providing a second opinion when needed. The data items extracted were as follows: type of review (term used by authors), aim of review (mapping evidence vs. answering specific question vs. borderline), number of visualisations (if any), types of data visualisation used, variables/domains presented by each visualisation type, interactivity, use of colour and any software requirements.

**Fig. 1:**
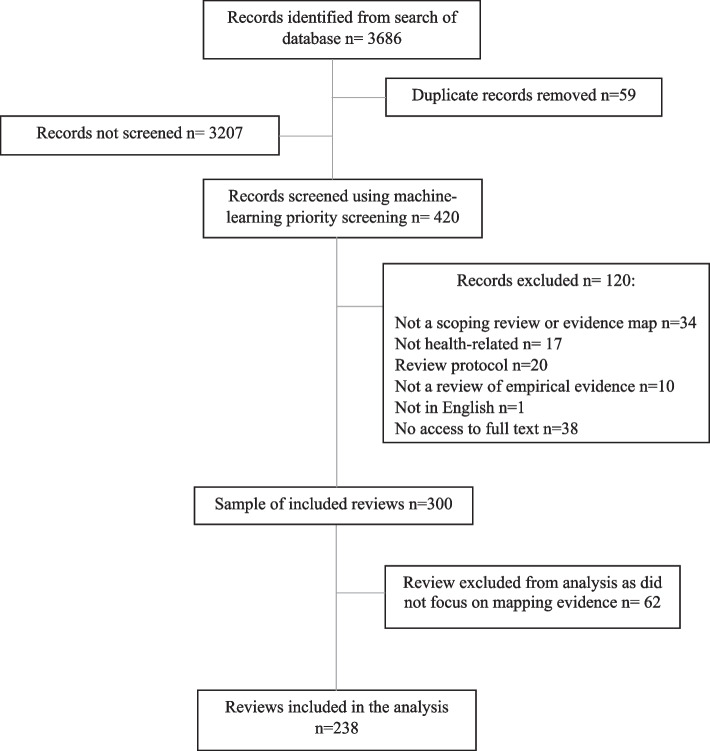
Flow diagram of the sampling process

When categorising review aims, we considered “mapping evidence” to incorporate all of the six purposes for conducting a scoping review proposed by Munn et al. [3]. Reviews were categorised as “answering a specific question” if they aimed to synthesise study findings to answer a particular question, for example on effectiveness of an intervention. We were inclusive with our definition of “mapping evidence” and included reviews with mixed aims in this category. However, some reviews were difficult to categorise (for example where aims were unclear or the stated aims did not match the actual focus of the paper) and were considered to be “borderline”. It became clear that a proportion of identified records that described themselves as “scoping” or “mapping” reviews were in fact pseudo-systematic reviews that failed to undertake key systematic review processes. Such reviews attempted to integrate the findings of included studies rather than map the evidence, and so reviews categorised as “answering a specific question” were excluded from the main analysis. Data visualisation methods for meta-analyses have been explored previously [19]. Figure 1 shows the flow of records from search results to final analysis sample.

Data visualisation was defined as any graph or diagram that presented results data, including tables with a visual mapping element, such as cross-tabulations and heat maps. However, tables which displayed data at a study level (e.g. tables summarising key characteristics of each included study) were not included, even if they used symbols, shading or colour. Flow diagrams showing the study selection process were also excluded. Data visualisations in appendices or supplementary information were included, as well as any in publicly available dissemination products (e.g. visualisations hosted online) if mentioned in papers.

The typology used to categorise data visualisation methods was based on an existing online catalogue [20]. Specific types of data visualisation were categorised in five broad categories: graphs, diagrams, tables, maps/geographical and other. If a data visualisation appeared in our sample that did not feature in the original catalogue, we checked a second online catalogue [21] for an appropriate term, followed by wider Internet searches. These additional visualisation methods were added to the appropriate section of the typology. The final typology can be found in Additional file 1.

### Analysis

We conducted descriptive data analysis in Microsoft Excel 2019 and present frequencies and percentages. Where appropriate, data are presented using graphs or other data visualisations created using Flourish. We also link to interactive versions of some of these visualisations.

## Results

Almost all of the 300 reviews in the total sample were labelled by review authors as “scoping reviews” (*n* = 293, 97.7%). There were also four “mapping reviews”, one “scoping study”, one “evidence mapping” and one that was described as a “scoping review and evidence map”. Included reviews were all published in 2020 or 2021, with the exception of one review published in 2018. Just over one-third of these reviews (*n* = 105, 35.0%) included some form of data visualisation. However, we excluded 62 reviews that did not focus on mapping evidence from the following analysis (see “Methods” section). Of the 238 remaining reviews (that either clearly aimed to map evidence or were judged to be “borderline”), 90 reviews (37.8%) included at least one data visualisation. The references for these reviews can be found in Additional file 2.

### Number of visualisations

**Fig. 2:**
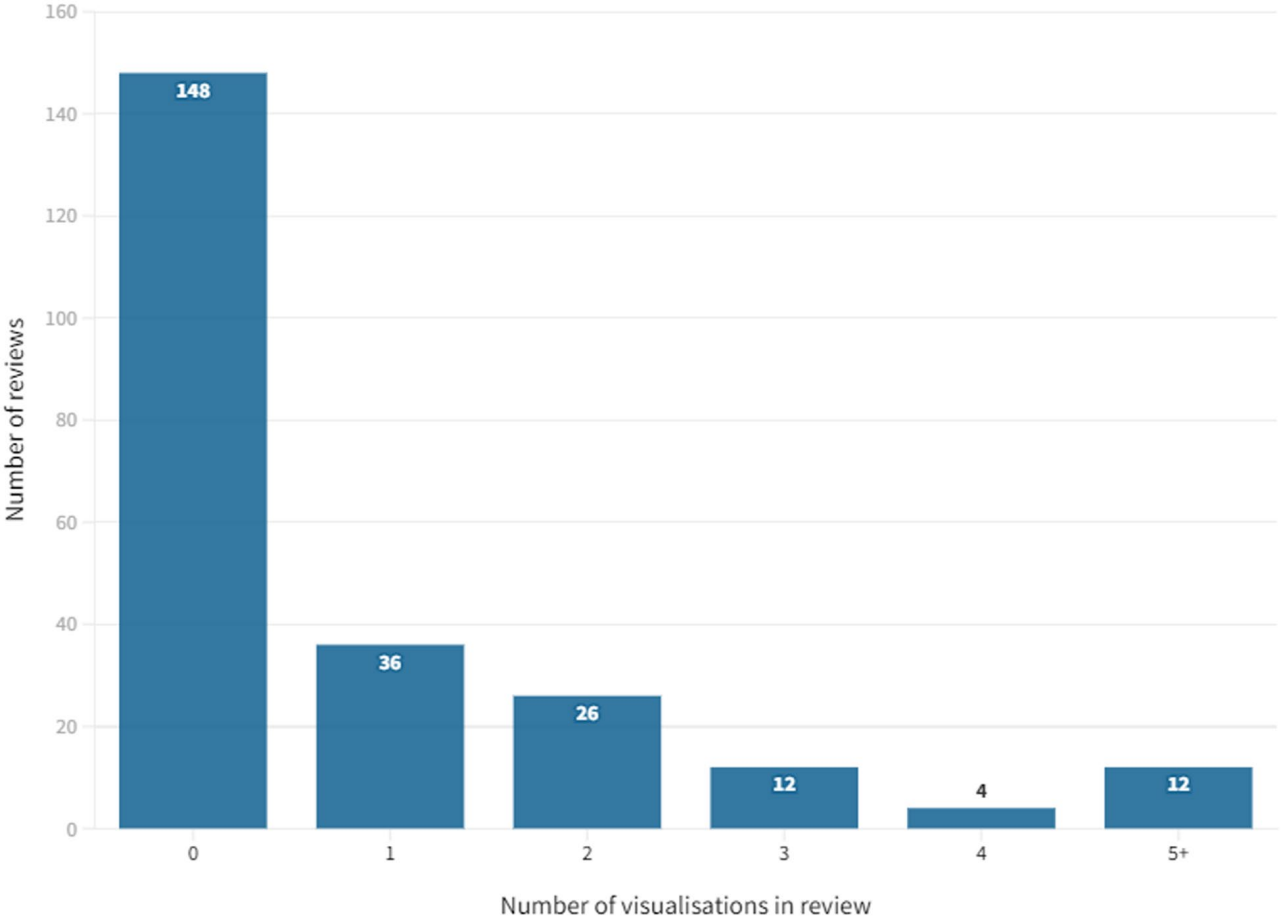
Number of data visualisations per review

Thirty-six (40.0%) of these 90 reviews included just one example of data visualisation (Fig. 2). Less than a third (*n* = 28, 31.1%) included three or more visualisations. The greatest number of data visualisations in one review was 17 (all bar or pie charts). In total, 222 individual data visualisations were identified across the sample of 238 reviews.

### Categories of data visualisation

Graphs were the most frequently used category of data visualisation in the sample. Over half of the reviews with data visualisation included at least one graph (*n* = 59, 65.6%). The least frequently used category was maps, with 15.6% (*n* = 14) of these reviews including a map.

Of the total number of 222 individual data visualisations, 102 were graphs (45.9%), 34 were tables (15.3%), 23 were diagrams (10.4%), 15 were maps (6.8%) and 48 were classified as “other” in the typology (21.6%).

### Types of data visualisation

All of the types of data visualisation identified in our sample are reported in Table 2. In total, 35 different types were used across the sample of reviews.

**Table 2:** Data visualisation types identified in sample of reviews

Data visualisation category and type	No. of data visualisations	%^a^	No. of reviews including this type	%^b^
**Graphs**	**102**	**45.9%**	**59**	**65.6%**
Bar chart	38	17.1%	26	28.9%
Stacked bar graph	27	12.2%	22	24.4%
Multi-set bar chart	13	5.9%	11	12.2%
Line graph	10	4.5%	7	7.8%
Histogram	4	1.8%	4	4.4%
Dot chart (with error bars)	6	2.7%	3	3.3%
Bubble chart	1	0.5%	1	1.1%
Scatterplot	1	0.5%	1	1.1%
Dumbbell plot	1	0.5%	1	1.1%
Butterfly chart	1	0.5%	1	1.1%
**Diagrams**	**23**	**10.4%**	**20**	**22.2%**
Concept map/framework/model	7	3.2%	6	6.7%
Network diagram	5	2.3%	4	4.4%
Timeline	3	1.4%	3	3.3%
Mind map	3	1.4%	2	2.2%
Tree diagram	2	0.9%	2	2.2%
Venn diagram	1	0.5%	1	1.1%
Alluvial diagram	1	0.5%	1	1.1%
Euler diagram	1	0.5%	1	1.1%
**Tables**	**34**	**15.3%**	**21**	**23.3%**
Cross-tabulation	24	10.8%	14	15.6%
Heat map	7	3.2%	4	4.4%
Matrix diagram	3	1.4%	3	3.3%
**Maps/geographical**	**15**	**6.8%**	**14**	**15.6%**
Choropleth map	6	2.7%	6	6.7%
Bubble map	4	1.8%	4	4.4%
Connection map	1	0.5%	1	1.1%
Pie chart(s) on map	1	0.5%	1	1.1%
Bubble × dot map^c^	1	0.5%	1	1.1%
Choropleth × dot map^d^	1	0.5%	1	1.1%
Choropleth × bubble map^e^	1	0.5%	1	1.1%
**Other**	**48**	**21.6%**	**24**	**26.7%**
Pie chart	33	14.9%	13	14.4%
Onion diagram	5	2.3%	5	5.6%
Word cloud	4	1.8%	3	3.3%
Proportional area chart	2	0.9%	2	2.2%
Sunburst diagram	2	0.9%	2	2.2%
Donut chart	1	0.5%	1	1.1%
Pictorial fraction chart	1	0.5%	1	1.1%

^a^% of total number of data visualisations. Some reviews included numerous examples of the same data visualisation type. ^b^% of 90 reviews with any data visualisation. Some reviews used multiple data visualisation types, so percentages do not total 100%. ^c^Data displayed through both dots and proportional circles. ^d^Data displayed through both colouring/shading and dots. ^e^Data displayed through both colouring/shading and proportional circles

**Fig. 3:**
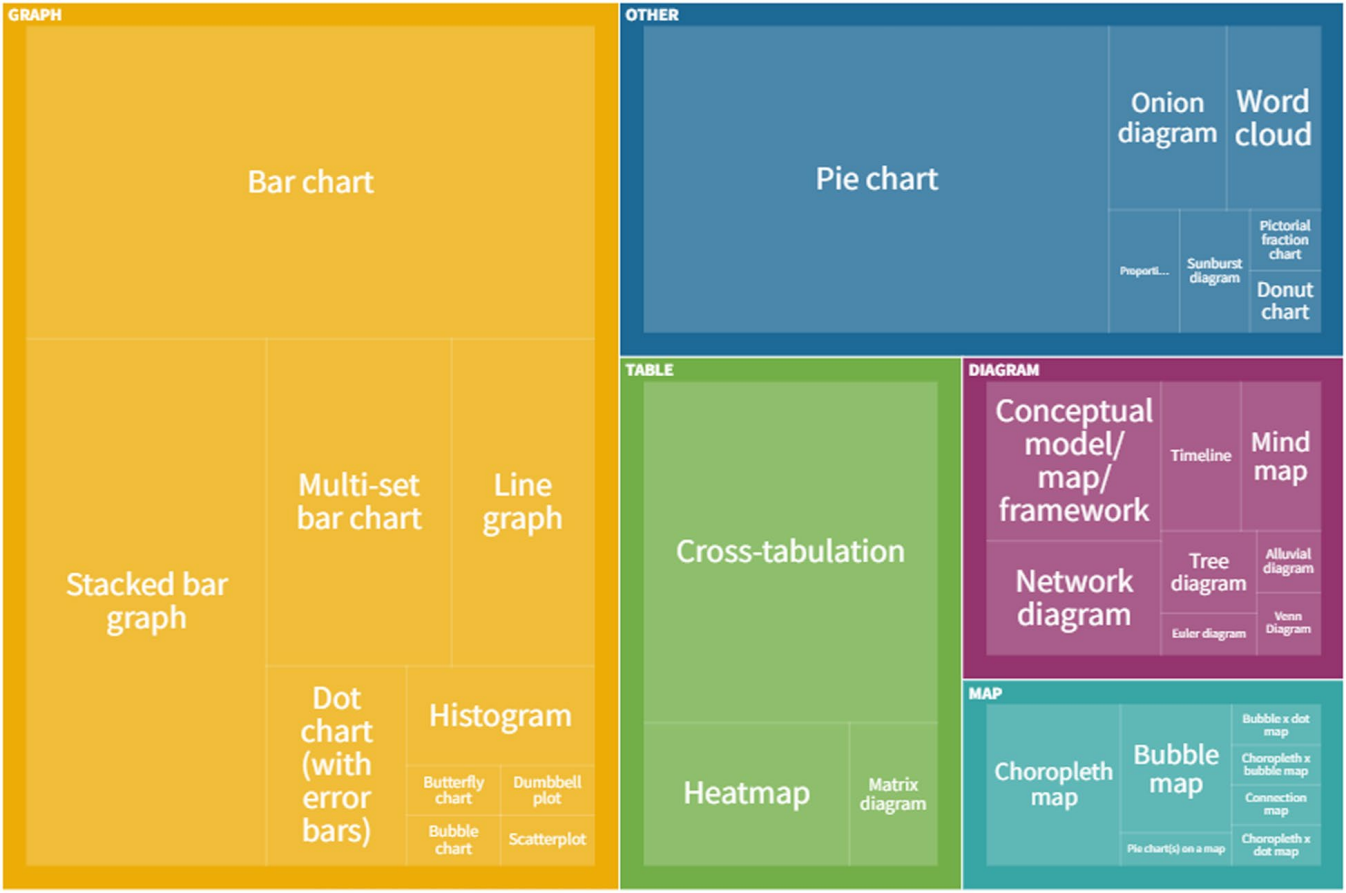
Data visualisation categories and types. An interactive version of this treemap is available online: https://public.flourish.studio/visualisation/9396133/. Through the interactive version, users can further explore the data (see Fig. 4). The unit of this treemap is the individual data visualisation, so multiple data visualisations within the same scoping review are represented in this map. Created with flourish.studio (https://flourish.studio)

**Fig. 4:**
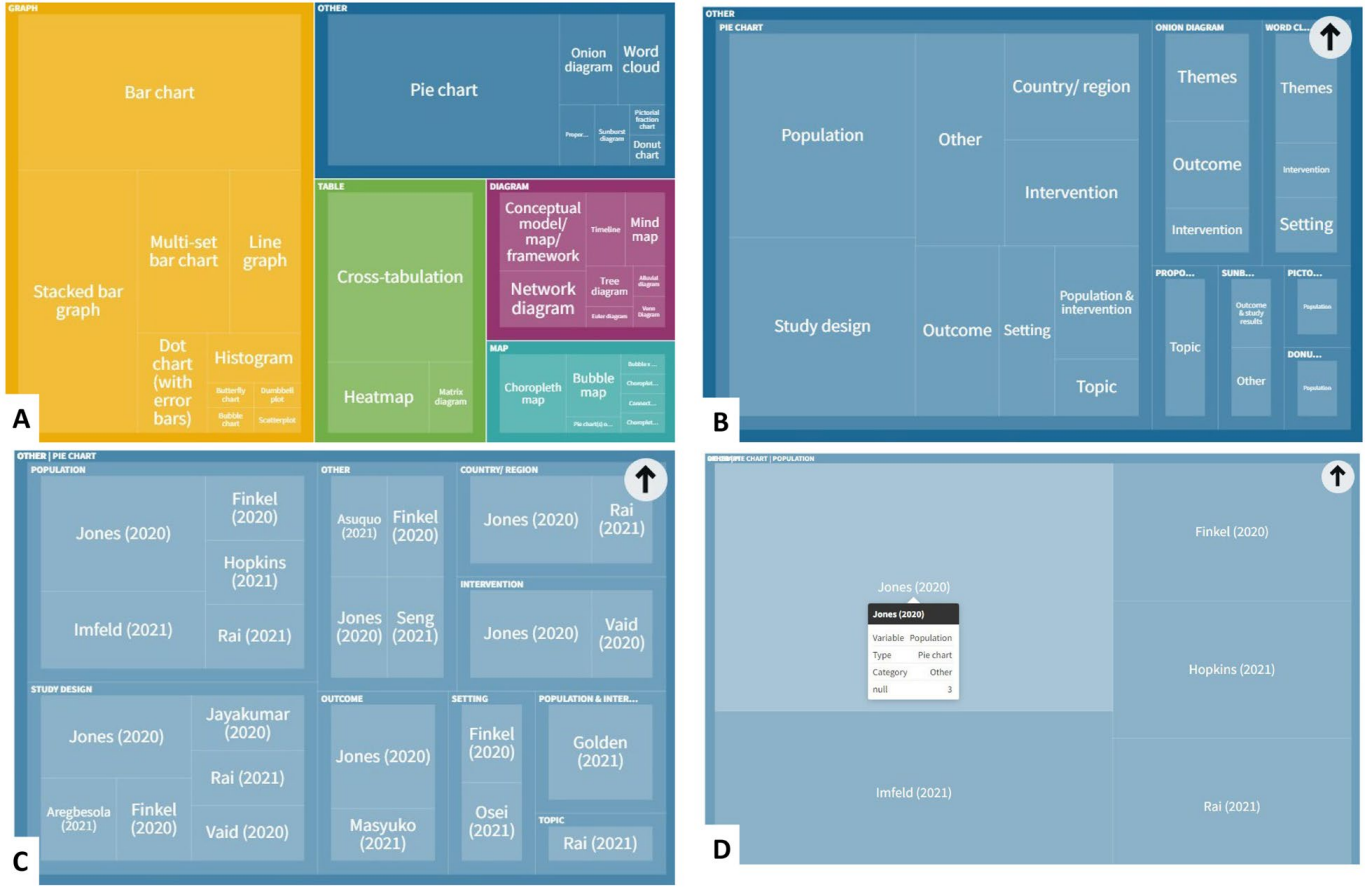
Screenshots showing how users of the interactive treemap can explore the data further. Users can explore each level of the hierarchical treemap (**A** Visualisation category > **B** Visualisation subcategory > **C** Variables presented in visualisation > **D** Individual references reporting this category/subcategory/variable permutation). Created with flourish.studio (https://flourish.studio)

The most frequently used data visualisation type was a bar chart. Of 222 total data visualisations, 78 (35.1%) were a variation on a bar chart (either standard bar chart, stacked bar chart or multi-set bar chart). There were also 33 pie charts (14.9% of data visualisations) and 24 cross-tabulations (10.8% of data visualisations). In total, these five types of data visualisation accounted for 60.8% (*n* = 135) of all data visualisations. Figure 3 shows the frequency of each data visualisation category and type; an interactive online version of this treemap is also available (https://public.flourish.studio/visualisation/9396133/). Figure 4 shows how users can further explore the data using the interactive treemap.

### Data presented

**Fig. 5:**
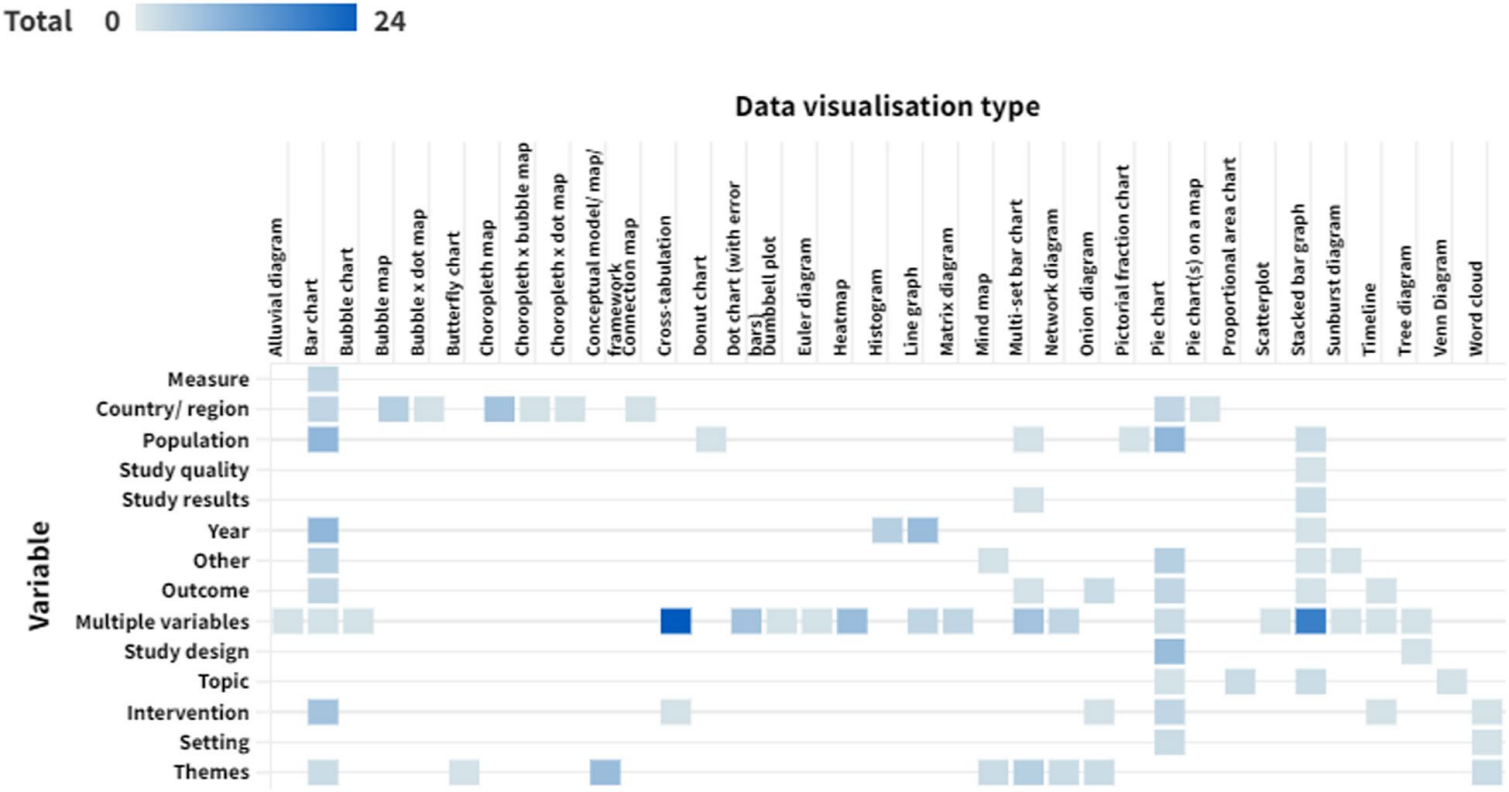
Variables presented by each data visualisation type. Darker cells indicate a larger number of reviews. An interactive version of this heat map is available online: https://public.flourish.studio/visualisation/10632665/. Users can hover over each cell to see the number of data visualisations for that combination of data visualisation type and variable. The unit of this heat map is the individual data visualisation, so multiple data visualisations within a single scoping review are represented in this map. Created with flourish.studio (https://flourish.studio)

Around two-thirds of data visualisations in the sample presented a single variable (*n* = 143, 64.4%). The most frequently presented single variables were themes (*n* = 22, 9.9% of data visualisations), population (*n* = 21, 9.5%), country or region (*n* = 21, 9.5%) and year (*n* = 20, 9.0%). There were 58 visualisations (26.1%) that presented two different variables. The remaining 21 data visualisations (9.5%) presented three or more variables. Figure 5 shows the variables presented by each different type of data visualisation (an interactive version of this figure is available online).

### Colour

Most reviews presented at least one data visualisation in colour (*n* = 64, 71.1%). However, almost a third (*n* = 26, 28.9%) used only black and white or greyscale.

### Interactivity

Only two of the reviews included data visualisations with any level of interactivity. One scoping review on music and serious mental illness [22] linked to an interactive bubble chart hosted online on Tableau. Functionality included the ability to filter the studies displayed by various attributes.

The other review was an example of evidence mapping from the environmental health field [23]. All four of the data visualisations included in the paper were available in an interactive format hosted either by the review management software or on Tableau. The interactive versions linked to the relevant references so users could directly explore the evidence base. This was the only review that provided this feature.

### Software requirements

Nine reviews clearly reported the software used to create data visualisations. Three reviews used Tableau (one of them also used review management software as discussed above) [22–24]. Two reviews generated maps using ArcGIS [25] or ArcMap [26]. One review used Leximancer for a lexical analysis [27]. One review undertook a bibliometric analysis using VOSviewer [28], and another explored citation patterns using CitNetExplorer [29]. Other reviews used Excel [30] or R [26].

## Discussion

To our knowledge, this is the first systematic and in-depth exploration of the use of data visualisation techniques in scoping reviews. Our findings suggest that the majority of scoping reviews do not use any data visualisation at all, and, in particular, more innovative examples of data visualisation are rare. Around 60% of data visualisations in our sample were simple bar charts, pie charts or cross-tabulations. There appears to be very limited use of interactive online visualisation, despite the potential this has for communicating results to a range of stakeholders. While it is not always appropriate to use data visualisation (or a simple bar chart may be the most user-friendly way of presenting the data), these findings suggest that data visualisation is being underused in scoping reviews. In a large minority of reviews, visualisations were not published in colour, potentially limiting how user-friendly and attractive papers are to decision-makers and other stakeholders. Also, very few reviews clearly reported the software used to create data visualisations. However, 35 different types of data visualisation were used across the sample, highlighting the wide range of methods that are potentially available to scoping review authors.

Our results build on the limited research that has previously been undertaken in this area. Two previous publications also found limited use of graphs in scoping reviews. Results were “mapped graphically” in 29% of scoping reviews in any field in one 2014 publication [31] and 17% of healthcare scoping reviews in a 2016 article [6]. Our results suggest that the use of data visualisation has increased somewhat since these reviews were conducted. Scoping review methods have also evolved in the last 10 years; formal guidance on scoping review conduct was published in 2014 [32], and an extension of the PRISMA checklist for scoping reviews was published in 2018 [33]. It is possible that an overall increase in use of data visualisation reflects increased quality of published scoping reviews. There is also some literature supporting our findings on the wide range of data visualisation methods that are used in evidence synthesis. An investigation of methods to identify, prioritise or display health research gaps (25/139 included studies were scoping reviews; 6/139 were evidence maps) identified 14 different methods used to display gaps or priorities, with half being “more advanced” (e.g. treemaps, radial bar plots) ([34], p. 107). A review of data visualisation methods used in papers reporting meta-analyses found over 200 different ways of displaying data [19].

Only two reviews in our sample used interactive data visualisation, and one of these was an example of systematic evidence mapping from the environmental health field rather than a scoping review (in environmental health, systematic evidence mapping explicitly involves producing a searchable database [35]). A scoping review of papers on the use of interactive data visualisation in population health or health services research found a range of examples but still limited use overall [13]. For example, the authors noted the currently underdeveloped potential for using interactive visualisation in research on health inequalities. It is possible that the use of interactive data visualisation in academic papers is restricted by academic publishing requirements; for example, it is currently difficult to incorporate an interactive figure into a journal article without linking to an external host or platform. However, we believe that there is a lot of potential to add value to future scoping reviews by using interactive data visualisation software. Few reviews in our sample presented three or more variables in a single visualisation, something which can easily be achieved using interactive data visualisation tools. We have previously used EPPI-Mapper [36] to present results of a scoping review of systematic reviews on behaviour change in disadvantaged groups, with links to the maps provided in the paper [37]. These interactive maps allowed policy-makers to explore the evidence on different behaviours and disadvantaged groups and access full publications of the included studies directly from the map.

We acknowledge there are barriers to use for some of the data visualisation software available. EPPI-Mapper and some of the software used by reviews in our sample incur a cost. Some software requires a certain level of knowledge and skill in its use. However numerous online free data visualisation tools and resources exist. We have used Flourish to present data for this review, a basic version of which is currently freely available and easy to use. Previous health research has been found to have used a range of different interactive data visualisation software, much of which does not required advanced knowledge or skills to use [13].

There are likely to be other barriers to the use of data visualisation in scoping reviews. Journal guidelines and policies may present barriers for using innovative data visualisation. For example, some journals charge a fee for publication of figures in colour. As previously mentioned, there are limited options for incorporating interactive data visualisation into journal articles. Authors may also be unaware of the data visualisation methods and tools that are available. Producing data visualisations can be time-consuming, particularly if authors lack experience and skills in this. It is possible that many authors prioritise speed of publication over spending time producing innovative data visualisations, particularly in a context where there is pressure to achieve publications.

### Limitations

A limitation of this study was that we did not assess how appropriate the use of data visualisation was in our sample as this would have been highly subjective. Simple descriptive or tabular presentation of results may be the most appropriate approach for some scoping review objectives [7, 8, 10], and the scoping review literature cautions against “over-using” different visual presentation methods [7, 8]. It cannot be assumed that all of the reviews that did not include data visualisation should have done so. Likewise, we do not know how many reviews used methods of data visualisation that were not well suited to their data.

We initially relied on authors’ own use of the term “scoping review” (or equivalent) to sample reviews but identified a relatively large number of papers labelled as scoping reviews that did not meet the basic definition, despite the availability of guidance and reporting guidelines [10, 33]. It has previously been noted that scoping reviews may be undertaken inappropriately because they are seen as “easier” to conduct than a systematic review ([3], p.6), and that reviews are often labelled as “scoping reviews” while not appearing to follow any established framework or guidance [2]. We therefore took the decision to remove these reviews from our main analysis. However, decisions on how to classify review aims were subjective, and we did include some reviews that were of borderline relevance.

A further limitation is that this was a sample of published reviews, rather than a comprehensive systematic scoping review as have previously been undertaken [6, 31]. The number of scoping reviews that are published has increased rapidly, and this would now be difficult to undertake. As this was a sample, not all relevant scoping reviews or evidence maps that would have met our criteria were included. We used machine learning to screen our search results for pragmatic reasons (to reduce screening time), but we do not see any reason that our sample would not be broadly reflective of the wider literature.

## Conclusions

Data visualisation, and in particular more innovative examples of it, is currently underused in published scoping reviews on health topics. The examples that we have found highlight the wide range of methods that scoping review authors could draw upon to present their data in an engaging way. In particular, we believe that interactive data visualisation has significant potential for mapping the available literature on a topic. Appropriate use of data visualisation may increase the usefulness, and thus uptake, of scoping reviews as a way of identifying existing evidence or research gaps by decision-makers, researchers and commissioners of research. We recommend that scoping review authors explore the extensive free resources and online tools available for data visualisation. However, we also think that it would be useful for publishers to explore allowing easier integration of interactive tools into academic publishing, given the fact that papers are now predominantly accessed online. Future research may be helpful to explore which methods are particularly useful to scoping review users.

### Supplementary Information

**Additional file 1.** Typology of data visualisation methods.

**Additional file 2.** References of scoping reviews included in main dataset.

## Data Availability

The datasets used and/or analysed during the current study are available from the corresponding author on reasonable request.

## Abbreviations

JBI: Organisation formerly known as Joanna Briggs Institute
PRISMA: Preferred Reporting Items for Systematic Reviews and Meta-Analyses
