# Supplementary material for: Data visualisation in scoping reviews and evidence maps on health topics: a cross-sectional analysis
Source: Syst Rev. 2023 Aug 17;12:142. doi: 10.1186/s13643-023-02309-y (PMC10433592; doi:10.1186/s13643-023-02309-y)
Supplement: Supplementary file 1 — Additional file 1. Typology of data visualisation methods. [file 13643_2023_2309_MOESM1_ESM.pdf]

Additional file 2: Typology of data visualisation methods

This is the full typology applied (and adapted) during data visualisation and therefore it includes data visualisation types that were not identified in the sample of reviews included in this study. It is adapted from: Severino Ribecca. The Data Visualisation Catalogue https://datavizcatalogue.com/ Accessed 22 November 2022.

- **Graphs**
  - Area graph
  - Bar chart
  - Box and whisker plot
  - Bubble chart
  - Bullet graph
  - Candlestick chart
  - Density plot
  - Histogram
  - Kagi chart
  - Line graph
  - Marimekko chart
  - Multi-set bar chart
  - Ohlc chart
  - Parallel coordinates plot
  - Point & figure chart
  - Population pyramid
  - Radar chart
  - Radial bar chart
  - Radial column chart
  - Scatterplot
  - Span chart
  - Spiral plot
  - Stacked area graph
  - Stacked bar graph
  - Stream graph
  - Violin plot

    - Dot chart
    - Dot chart (with error bars)
    - Forest plot
    - Dumbell plot
    - Jitter plot
    - Butterfly chart

- **Diagrams**
    - Arc diagram
    - Brainstorm
    - Chord diagram
    - Flow chart
    - Illustration diagram
    - Network diagram
    - Non-ribbon chord diagram
    - Sankey diagram
    - Timeline
    - Tree diagram
    - Venn diagram
    - Concept map/framework/ model
    - Alluvial diagram
    - Euler diagram
    - Bubble timeline

- **Tables**
    - Calendar
    - Gantt chart
    - Heatmap
    - Stem & leaf plot
    - Tally chart
    - Time table
    - Cross-tabulation

    - Matrix diagram

- **Maps/geographical**
    - Bubble map
    - Choropleth map
    - Connection map
    - Dot map
    - Flow map
    - Pie chart(s) on map
    - Bubble x dot map
    - Choropleth x dot map
    - Choropleth x bubble map

- **Other**
    - Circle packing
    - Donut chart
    - Dot matrix chart
    - Nightingale rose chart
    - Parallel sets
    - Pictogram chart
    - Pie chart
    - Proportional area chart
    - Sunburst diagram
    - Treemap
    - Word cloud
    - Onion diagram
    - Pictorial fraction chart
    - Other (unknown type)
