# Supplementary material for: Data visualisation in scoping reviews and evidence maps on health topics: a cross-sectional analysis
Source: Syst Rev. 2023 Aug 17;12:142. doi: 10.1186/s13643-023-02309-y (PMC10433592; doi:10.1186/s13643-023-02309-y)
Supplement: Supplementary file 2 — Additional file 2. References of scoping reviews included in main dataset. [file 13643_2023_2309_MOESM2_ESM.pdf]

Additional file 2: References of scoping reviews included in main dataset (scoping reviews that included data visualisation and had an aim of mapping)

1. Aapro M, Bossi P, Dasari A, Fallowfield L, Gascon P, Geller M, et al. (2020). Digital health for optimal supportive care in oncology: benefits, limits, and future perspectives. *Supportive Care in Cancer*, 28, pp.4589-4612.
2. Ambler M, Springs S, Garcia D, Born C. (2021). Heterogeneity of outcomes for intraoperative music interventions: a scoping review and evidence map. *BMJ Evidence-Based Medicine*, 26, pp.116-117.
3. Aregbesola A, Abou-Setta A M, Okoli G N, Jeyaraman M M, Lam O, Kasireddy V et al. (2021). Implementation strategies in emergency management of children: A scoping review. *PloS One*, 16, pp.e0248826.
4. Asuquo S E, Tahlil K M, Muessig K E, Conserve D F, Igbokwe M A, Chima K P, et al. (2021). Youth engagement in HIV prevention intervention research in sub-Saharan Africa: a scoping review. *Journal of the International AIDS Society*, 24, pp.e25666.
5. Bedard A, Lamarche P, Gregoire L, Trudel-Guy C, Provencher V, Desroches S, et al. (2020). Can eating pleasure be a lever for healthy eating? A systematic scoping review of eating pleasure and its links with dietary behaviors and health. *PloS One*, 15, pp.e0244292.
6. Beldhuis I E, Marapin R S, Jiang Y Y, Simoes de Souza N F, Georgiou A, Kaufmann T, et al. (2021). Cognitive biases, environmental, patient and personal factors associated with critical care decision making: A scoping review. *Journal of Critical Care*, 64, pp.144-153.
7. Boyd L A P, Waller A E, Hill D, Sanson-Fisher R W. (2021). Psychosocial well-being of brain cancer patients and support persons: A mapping review of study types over time. *European Journal of Cancer Care*, 30, pp.e13446.
8. Buchanan H, Van Niekerk L, Grimmer. (2020). Work transition after hand injury: A scoping review. *Journal of Hand Therapy*, 35 pp.11-23.
9. Cai K, Fuller A, Hensey O, Grossberg D, Christensen R, Shea B et al. (2020). Outcome domains reported in calcium pyrophosphate deposition studies: A scoping review by the OMERACT CPPD working group. *Seminars in Arthritis and Rheumatism*, 50, pp.719-727.
10. Challa S, Agarwal-Harding K J, Levy P, Barr-Walker J, Sabatini Coleen S. (2020). Supracondylar humerus fractures in low- and lower middle-income countries: a scoping review of the current epidemiology, treatment modalities, and outcomes. *International Orthopaedics*, 44, pp.2443-2448.
11. Chao G F, Emlaw J, Chiu A S, Yang J, Thumma J, Brackett A, et al. (2021). Asian American Pacific Islander representation in outcomes research: NSQIP scoping review. *Journal of the American College of Surgeons*, 232, pp.682-689.e5.
12. Cherak S J, Rosgen B K, Amarbayan M, Plotnikoff K, Wollny K, Stelfox H T, et al. (2020). Impact of social media interventions and tools among informal caregivers of critically ill patients after patient admission to the intensive care unit: A scoping review. *PloS One*, 15, pp.e0238803.
13. Elshahat S, Treanor C, Donnelly M. (2021). Factors influencing physical activity participation among people living with or beyond cancer: a systematic scoping review. *The International Journal of Behavioral Nutrition and Physical Activity*, 18, pp.50.
14. Fakha A, Groenvynck L, de Boer B, van Achterberg T, Hamers J, Verbeek H, (2021). A myriad of factors influencing the implementation of transitional care innovations: a scoping review. *Implementation Science*, 16, pp.21.
15. Finkel D, Bat Or M. (2020). The open studio approach to art therapy: a systematic scoping review. *Frontiers in Psychology*, 11, pp.568042.

16. Giusepi I, St John A, Julicher P. (2020). Who conducts health economic evaluations of laboratory tests? a scoping review. *The Journal of Applied Laboratory Medicine*, 5, pp.954-966.
17. Giusti E M, Manna C, Scolari A, Mestre J M, Prevendar T, Castelnuovo G, et al. (2021). The relationship between emotional intelligence, obesity and eating disorder in children and adolescents: a systematic mapping review. *International Journal of Environmental Research and Public Health*, 18, pp.2054.
18. Golden T L, Springs S, Kimmel H J, Gupta S, Tiedemann A, Sandu C C, et al, (2021). The use of music in the treatment and management of serious mental illness: a global scoping review of the literature. *Frontiers in Psychology*, 12, pp.649840.
19. Golding H, Webber C E, Groome P A. (2021). Factors contributing to time to diagnosis in symptomatic colorectal cancer: a scoping review. *European Journal of Cancer Care*, 30, pp.e13397.
20. Gomez-Rossi J, Hertrampf K, Abraham J, Gasmann G, Meyer G, Schlattmann P, et al. (2020). Interventions to improve oral health of older people: A scoping review. *Journal of Dentistry*, 101, pp.103451.
21. Gonzalez J N, Axiotakis, Jr L G, Yu V X, Gudis D A, Overdevest J B. (2021). Practice of telehealth in otolaryngology: a scoping review in the era of COVID-19. *Otolaryngology--Head and Neck Surgery*, 166, pp.1945998211013751.
22. Graetz D E, Garza M, Rodriguez-Galindo C, Mack, JW. (2020). Pediatric cancer communication in low- and middle-income countries: a scoping review. *Cancer*, 126, pp.5030-5039.
23. Griswold D P, Fernandez L, Rubiano A M. (2021). Traumatic subarachnoid hemorrhage: a scoping review. *Journal of Neurotrauma*, 39, pp.35-38.
24. Hewitt L, Dahlen H G, Hartz D L, Dadich A. (2021). Leadership and management in midwifery-led continuity of care models: a thematic and lexical analysis of a scoping review. *Midwifery*, 98, pp.102986.
25. Holt C J, McKay C D, Truong L K, Le C Y, Gross D P, Whittaker J L. (2020). Sticking to it: a scoping review of adherence to exercise therapy interventions in children and adolescents with musculoskeletal conditions. *The Journal of Orthopaedic and Sports Physical Therapy*, 50, pp.503-515.
26. Hoover J, Koon A D, Rosser E N, Rao K D. (2020). Mentoring the working nurse: a scoping review. *Human Resources for Health*, 18, pp.52.
27. Hopkins H, Weaks C and Napier E. (2021). Implementation and utilization of gynecological teaching associate and male urogenital teaching associate programs: a scoping review. *Advances in Simulation*, 6, pp.19.
28. Huter K, Krick T, Domhoff D, Seibert K, Wolf-Ostermann K, Rothgang H. (2020). Effectiveness of digital technologies to support nursing care: results of a scoping review. *Journal of Multidisciplinary Healthcare*, 13, pp.1905-1926.
29. Imfeld S M, Darang D M, Neudecker M, McVoy M K. (2021). Primary care pediatrician perceptions towards mental health within the primary care setting. *Pediatric Research*, 90, pp.950-956.
30. Jayakumar P, Lin E, Galea V, Mathew A J, Panda N, Vetter I, et al. (2020). Digital phenotyping and patient-generated health data for outcome measurement in surgical care: a scoping review. *Journal of Personalized Medicine*, 10, pp.282.
31. Jimenez G, Spinazze P, Matchar D, Koh Choon Huat G, van der Kleij R M J J, Chavannes N H, et al. (2020). Digital health competencies for primary healthcare professionals: a scoping review. *International Journal of Medical Informatics*, 143, pp.104260.

32. Johnson L, Gutridge K, Parkes J, Roy A, Plugge E. (2021). Scoping review of mental health in prisons through the COVID-19 pandemic. *BMJ Open*, 11, pp.e046547.
33. Jones C, Miguel-Cruz A, Smith-MacDonald L, Cruikshank E, Baghoori D, Kaur C, et al. (2020). Virtual trauma-focused therapy for military members, veterans, and public safety personnel with posttraumatic stress injury: systematic scoping review. *JMIR mHealth and uHealth*, 8, pp.e22079.
34. Jones C M, Gautier L, Ridde V. (2021). A scoping review of theories and conceptual frameworks used to analyse health financing policy processes in sub-Saharan Africa. *Health Policy and Planning*, 36, pp.1197-1214.
35. Kapadia M R, Lee E, Healy H, Dort J M, Rosenbaum M E, Newcomb A B. (2021). Training surgical residents to communicate with their patients: a scoping review of the literature. *Journal of Surgical Education*, 78, pp.440-449.
36. Kaur P, Tan W S, Gunapal P P G, Ding Y Y, Ong, Wu H Y, et al. (2020). Deaths in dementia: a scoping review of prognostic variables. *BMJ Supportive & Palliative care*, 11, pp.242-252.
37. Kersting C, Kneer M, Barzel A. (2020). Patient-relevant outcomes: what are we talking about? A scoping review to improve conceptual clarity. *BMC Health Services Research*, 20, pp.596.
38. Keshava C, Davis J A, Stanek J, Thayer K A, Galizia A, Keshava N, et al. (2020). Application of systematic evidence mapping to assess the impact of new research when updating health reference values: A case example using acrolein. *Environment International*, 143, pp.105956.
39. Krishnaratne S, Pfadenhauer L M, Coenen M, Geffert K, Jung-Sievers C, Klinger C, et al. (2020). Measures implemented in the school setting to contain the COVID-19 pandemic: a scoping review. *The Cochrane Database of Systematic Reviews*, 12, pp.CD013812.
40. Lim X Y, Tan T Y C, Rosli S H M, Sa'at M N F, Ali S S, Mohamed A F S. (2021). Cannabis sativa subsp. sativa's pharmacological properties and health effects: A scoping review of current evidence. *PloS One*, 16, pp.e0245471.
41. Linn N, Goetzinger C, Regnaux J, Schmitz S, Dessenne C, Fagherazzi G, et al. (2021). Digital health interventions among people living with frailty: a scoping review. *Journal of the American Medical Directors Association*, 22, pp.1802-1812.
42. Martiniello N, Wittich W. (2020). The association between tactile, motor and cognitive capacities and braille reading performance: a scoping review of primary evidence to advance research on braille and aging. *Disability and Rehabilitation*, 44, pp.2515-2536.
43. Masyuko S, Ngongo C J, Smith C, Nugent R. (2021). Patient-reported outcomes for diabetes and hypertension care in low- and middle-income countries: A scoping review. *PloS One*, 16, pp.e0245269.
44. McNett M, O'Mathuna D, Tucker S, Roberts H, Mion L C, Balas MC. (2020). A scoping review of implementation science in adult critical care settings. *Critical Care Explorations*, 2, pp.e0301.
45. Migliore L, Braun L, Stucky C H, Gardner C, Huffman S, Jumpp S, et al. (2020). Considerations for acute and emergent deployed mental health patient management and theater transports: a scoping review. *Military Medicine*, 186, pp.e932-e942.
46. Miller K, O'Hara N N, Welsh C J, Ordonio K, Loughry N, Liu L, et al. (2018). Themes and gaps in research for opioid use and misuse pertinent to orthopaedic injury patients. *OTA International: the Open Access Journal of Orthopaedic Trauma*, 1, pp.e002.
47. Muller A E, Tveito K, Bakken I J, Flottorp S A, Mjaaland S, Larun L. (2020). Potential causal factors of CFS/ME: a concise and systematic scoping review of factors researched. *Journal of Translational Medicine*, 18, pp.484.

48. Nawrath M, Guenat S, Elsey H, Dallimer M. (2021). Exploring uncharted territory: Do urban greenspaces support mental health in low- and middle-income countries?. *Environmental Research*, 194, pp.110625.
49. Nelson N R, Carlson R B, Corbett A H, Williams D M, Rhoney D H. (2021). Feedback for learning in pharmacy education: a scoping review. *Pharmacy*, 9, pp.91.
50. Nilsson M Y, Andersson S, Magnusson L, Hanson E. (2021). Ambient assisted living technology-mediated interventions for older people and their informal carers in the context of healthy ageing: a scoping review. *Health Science Reports*, 4, pp.e225.
51. Nino de Guzman Quispe E, Martinez Garcia L, Orrego Villagran C, Heijmans M, Sunol R, Fraile-Navarro D, et al. (2021). The perspectives of patients with chronic diseases and their caregivers on self-management interventions: a scoping review of reviews. *The Patient*, 14, pp.719-740.
52. Nnaji C A, Wiysonge C S, Okeibunor J C, Malinga T, Adamu A A, Tumusiime P, et al. (2021). Implementation research approaches to promoting universal health coverage in Africa: a scoping review. *BMC Health Services Research*, 21, pp.414.
53. Osei E, Kuupiel D, Vezi P N, Mashamba-Thompson T P. (2021). Mapping evidence of mobile health technologies for disease diagnosis and treatment support by health workers in sub-Saharan Africa: a scoping review. *BMC Medical Informatics and Decision Making*, 21, pp.11.
54. Plante J, Latulippe K, Kroger E, Giroux D, Marcotte M, Nadeau S, et al. (2021). Cognitive impairment and length of stay in acute care hospitals: a scoping review of the literature. *Canadian Journal on Aging*, 40, pp.1-19.
55. Pratt M, Garritty C, Thuku M, Esmaeilisaraji L, Hamel C, Hartley T, et al. (2020). Application of exome sequencing for prenatal diagnosis: a rapid scoping review. *Genetics in Medicine*, 22, pp.1925-1934.
56. Putri L P, O'Sullivan B G, Russell D J, Kippen R. (2020). Factors associated with increasing rural doctor supply in Asia-Pacific LMICs: a scoping review. *Human Resources for Health*, 18, pp.93.
57. Qu L G, Perera M, Lawrentschuk N, Umbas R, Klotz L. (2020). Scoping review: hotspots for COVID-19 urological research: what is being published and from where?. *World Journal of Urology*, 39, pp.3151-3160.
58. Rahn A C, Solari A, Beckerman H, Nicholas R, Wilkie D, Heesen C, et al. (2020). "I will respect the autonomy of my patient": a scoping review of shared decision making in multiple sclerosis. *International Journal of MS Care*, 22, pp.285-293.
59. Rai R, El-Zaemey S, Dorji N, Rai B D, Fritschi L. (2021). Exposure to occupational hazards among health care workers in low- and middle-income countries: a scoping review. *International Journal of Environmental Research and Public health*, 18, pp.2603.
60. Ramji M, Steve A K, Premji Z, Yeung J. (2020). Functional outcomes of major upper extremity replantation: a scoping review. *Plastic and reconstructive surgery. Global Open*, 8, pp.e3071.
61. Riley I L, Jackson B, Crabtree D, Riebl S, Que L G, Pleasants R, et al. (2021). A scoping review of international barriers to asthma medication adherence mapped to the theoretical domains framework. *The Journal of Allergy and Clinical Immunology. In Practice*, 9, pp.410-418.e4.
62. Rossa-Roccor V, Acheson E S, Andrade-Rivas F, Coombe M, Ogura S, Super L, et al. (2020). Scoping review and bibliometric analysis of the term "planetary health" in the peer-reviewed literature. *Frontiers in Public Health*, 8, pp.343.

63. Samadbeik M, Fatehi F, Braunstein M, Barry B, Saremian M, Kalhor F, et al. (2020). Education and training on electronic medical records (EMRs) for health care professionals and students: a scoping review. *International Journal of Medical Informatics*, 142, pp.104238.
64. Schiffer V M M M, Janssen E B N J, van Bussel B C T, Jorissen L L M, Tas J, Sels J E M, et al. (2020). The "sex gap" in COVID-19 trials: a scoping review. *EClinicalMedicine*, 29, pp.100652.
65. Seng J J B, Monteiro A Y, Kwan Y H, Zainudin S B, Tan C S, Thumboo J, et al. (2021). Population segmentation of type 2 diabetes mellitus patients and its clinical applications - a scoping review. *BMC Medical Research Methodology*, 21, pp.49.
66. Shinozaki N, Yuan X, Murakami K, Sasaki S. (2021). Development, validation and utilisation of dish-based dietary assessment tools: a scoping review. *Public Health Nutrition*, 24, pp.223-242.
67. Sigfrid L, Maskell K, Bannister P G, Ismail S A, Collinson S, Regmi S, et al. (2020). Addressing challenges for clinical research responses to emerging epidemics and pandemics: a scoping review. *BMC Medicine*, 18, pp.190.
68. Smith V, Devane D, Nichol A, Roche D. (2020). Care bundles for improving outcomes in patients with COVID-19 or related conditions in intensive care - a rapid scoping review. *The Cochrane Database of Systematic Reviews*, 12, pp.CD013819.
69. Smith M K, Xu R H, Hunt S L, Wei C, Tucker J D, Tang W, et al. (2020). Combating HIV stigma in low- and middle-income healthcare settings: a scoping review. *Journal of the International AIDS Society*, 23, pp.e25553.
70. Soler F, Mocini F, Djemeto D T, Cattaneo S, Saccomanno M F, Milano G. (2021). No differences between conservative and surgical management of acromioclavicular joint osteoarthritis: a scoping review. *Knee Surgery, Sports Traumatology and Arthroscopy*, 29, pp.2194-2201.
71. Spielmann H, Seemann M, Friedrich N, Tigges-Limmer K, Albert W, Semmig-Konze S, et al. (2021). Self-management with the therapeutic regimen in patients with ventricular assist device (VAD) support - a scoping review. *Heart & Lung: The Journal of Critical Care*, 50, pp.388-396.
72. Sprow H N, Hansen N F, Loeb H E, Wight Ce L, Patterson R H, Vervoort D et al. (2021). Gender-based microaggressions in surgery: a scoping review of the global literature. *World Journal of Surgery*, 45, pp.1409-1422.
73. Sugden N, Thomas M, Kiernan M. (2021). A scoping review of the utility of self-report and informant-report prospective memory measures. *Neuropsychological Rehabilitation*, , pp.1-31.
74. Tirado V, Chu J, Hanson C, Ekstrom A M, Kagesten A. (2020). Barriers and facilitators for the sexual and reproductive health and rights of young people in refugee contexts globally: A scoping review. *PloS One*, 15, pp.e0236316.
75. Tsai C H, Eghdam A, Davoody N, Wright G, Flowerday S, Koch S. (2020). Effects of electronic health record implementation and barriers to adoption and use: a scoping review and qualitative analysis of the content. *Life*, 10, pp.327.
76. Vaid N R, Hansa I, Bichu Y. (2020). Smartphone applications used in orthodontics: a scoping review of scholarly literature. *Journal of the World Federation of Orthodontists*, 9, pp.S67-S73.
77. van Heemskerken P, Broekhuizen H, Gajewski J, Brugha R, Bijlmakers L. (2020). Barriers to surgery performed by non-physician clinicians in sub-Saharan Africa- a scoping review. *Human Resources for Health*, 18, pp.51.

78. Vigliotti V, Taggart T, Walker M, Kusmastuti S, Ransome Y. (2020). Religion, faith, and spirituality influences on HIV prevention activities: a scoping review. *PloS One*, 15, pp.e0234720.
79. Voss M, Swart O, Abel L, Mahtani K. (2021). Capacity-building partnerships for surgical post-graduate training in low- and middle-income countries: a scoping review of the literature with exploratory thematic synthesis. *Health Policy and Planning*, 35, pp.1385-1412.
80. Wake E, Atkins H, Willock A, Hawkes A, Dawber J, Weir K A. (2020). Telehealth in trauma: a scoping review. *Journal of Telemedicine and Telecare*, 28, pp.1357633X20940868.
81. Walcott S E, Miller F A, Dunsmore K, Lazor T, Feldman B M, Hayeems R Z. (2021). Measuring clinical utility in the context of genetic testing: a scoping review. *European Journal of Human Genetics*, 29, pp.378-386.
82. Wali S, Superina S, Mashford-Pringle A, Ross H, Cafazzo J A. (2021). What do you mean by engagement? - evaluating the use of community engagement in the design and implementation of chronic disease-based interventions for Indigenous populations - scoping review. *International Journal for Equity in Health*, 20, pp.8.
83. Walker A, Hing W, Lorimer A. (2020). The influence, barriers to and facilitators of anterior cruciate ligament rehabilitation adherence and participation: a scoping review. *Sports Medicine* - *Open*, 6, pp.32.
84. Wang L, Norman I, Xiao T, Li Y, Leamy M. (2021). Psychological first aid training: a scoping review of its application, outcomes and implementation. *International Journal of Environmental Research and Public health*, 18, pp.4594.
85. Whitmore K A, Townsend S C, Laupland K B. (2020). Management of tracheostomies in the intensive care unit: a scoping review. *BMJ Open Respiratory Research*, 7, pp.e000651.
86. Wild T C, Hammal F, Hancock M, Bartlett N T, Gladwin K K, Adams D, et al. (2021). Forty-eight years of research on psychosocial interventions in the treatment of opioid use disorder: a scoping review. *Drug and Alcohol Dependence*, 218, pp.108434.
87. Wilkes J R, Walter A E, Chang A, Miller S J, Sebastianelli W J, Seidenberg P H, et al. (2021). Effects of sleep disturbance on functional and physiological outcomes in collegiate athletes: a scoping review. *Sleep Medicine*, 81, pp.8-19.
88. Xepoleas M D, Munabi N C O, Auslander A, Magee W, P, Yao C A. (2020). The experiences of female surgeons around the world: a scoping review. *Human Resources for Health*, 18, pp.80.
89. Xia H, Tan S, Huang S, Gan P, Zhong C, Lu M, et al. (2021). Scoping review and bibliometric analysis of the most influential publications in achalasia research from 1995 to 2020. *BioMed research International*, 2021, pp.8836395.
90. Zhao Y, Musitia P, Boga M, Gathara D, Nicodemo C, English M. (2021). Tools for measuring medical internship experience: a scoping review. *Human Resources for Health*, 19, pp.10.
